# Ethics challenges and guidance related to research involving adolescent post-abortion care: a scoping review

**DOI:** 10.1186/s12978-018-0515-6

**Published:** 2018-05-02

**Authors:** Joseph M. Zulu, Joseph Ali, Kristina Hallez, Nancy Kass, Charles Michelo, Adnan A. Hyder

**Affiliations:** 1 School of Public Health, University of Zambia, P.O. Box 50110, Lusaka, Zambia; 2 Johns Hopkins Berman Institute of Bioethics, Deering Hall, 1809 Ashland Avenue, Baltimore, MD 21205 USA; 3 Department of International Health, Johns Hopkins Bloomberg School of Public Health, 615 N. Wolfe Street, Baltimore, MD 21205 USA

**Keywords:** Post abortion care, Research ethics, Adolescents, Ethics guidance, Reproductive ethics

## Abstract

**Introduction:**

An increase in post abortion care (PAC) research with adolescents, particularly in low- and middle-income countries, has brought to attention several associated research ethics challenges. In order to better understand the ethics context of PAC research with adolescents, we conducted a scoping review of published literature.

**Methods:**

Following a systematic search of PubMed, HINARI, and Google Scholar, we analysed articles meeting inclusion criteria to determine common themes across both the ethical challenges related to PAC research with adolescents and any available guidance on the identified challenges.

**Results:**

The literature search identified an initial 3321 records of which 14 were included in analysis following screening. Several ethical challenges stem from abortion being a controversial, sensitive, and stigmatized topic in many settings. Ethical dilemmas experienced by researchers conducting adolescent PAC research included: difficulties in convincing local health providers to permit PAC research; challenges in recruiting and seeking consent due to sensitivity of the subject; effectively protecting confidentiality; managing negative effects of interventions; creating a non-prejudicial atmosphere for research; managing emotional issues among adolescents; and dealing with uncertainty regarding the role of researchers when observing unethical health care practices. Suggested strategies for addressing some of these challenges include: using several sources to recruit study participants, using research to facilitate dialogue on abortion, briefing health workers on any observed unethical practices after data collection, fostering a comprehensive understanding of contextual norms and values, selecting staff with experience working with study populations, and avoiding collection of personal identifiers.

**Conclusion:**

Addressing ethical challenges that researchers face when conducting PAC research with adolescents requires guidance at the individual, institutional, community, and international levels. Overall, despite the documentation of challenges in the published literature, guidance on handling several of these ethics challenges is sparse. We encourage further research to clarify the identified challenges and support the development of formal guidance in this area.

## Plain English summary

We conducted a scoping review of published literature in order to better understand the ethics challenges associated with post abortion care (PAC) research with adolescents in low and middle in countries.

We systematically searched PubMed, HINARI, and Google Scholar to identify relevant articles from which we analysed both the ethical challenges related to PAC research with adolescents and any available guidance on the challenges.

Fourteen articles were included in the final analysis. Several ethical challenges were identified and these included difficulties in convincing local health providers to permit PAC research; challenges in recruiting and seeking consent due to sensitivity of the subject; as well as difficulties in effectively protecting confidentiality; managing negative effects of interventions; creating a non-prejudicial atmosphere for research; managing emotional issues among adolescents; and dealing with uncertainty regarding the role of researchers when observing unethical health care practices. These challenges were addressed through using several sources to recruit study participants, briefing health workers on any observed unethical practices after data collection, fostering a comprehensive understanding of contextual norms and values, selecting staff with experience working with study populations, and avoiding collection of personal identifiers.

In conclusion, it is important that proper guidance is provided at the individual, institutional, community, and international levels if the ethical challenges that researchers face when conducting PAC research with adolescents are to be addressed.

## Background

Approximately 25 million unsafe abortions occurred annually worldwide between 2010 and 2014 [1]. Almost 97% of the unsafe abortions occurred in developing countries in Africa, Asia and Latin America [1]. Globally, each year between 4.7% – 13.2% of maternal deaths result from unsafe abortion [2]. In developing countries, about 7 million women are admitted to hospitals every year as a result of unsafe abortion [1–3]. In Sub-Saharan Africa, the contribution of unsafe abortion to maternal death is as high as 30% [3]. Unsafe abortion can also results in complications such as chronic pain and secondary infertility [1–3]. In Zambia in particular, maternal mortality is high at 398/100,000 live births [4], 30% of these deaths are caused by unsafe abortion and 80% involve adolescents [5, 6]. These numbers may underestimate impact as many adolescents do not seek care in hospitals [5, 6].

Post abortion care (PAC) seeks to help address abortion-related complications [7, 8]. In 1991, PAC was articulated as a critical component of women’s health initiatives by the PAC Consortium [9], consisting of organisations and individuals that work on and are interested in PAC, and support prevention, treatment, and counselling services to respond to sexual and reproductive health (SRH) needs and concerns of women. The Essential Elements of the PAC model incorporates the following: 1) Community and service provider partnerships; 2) Counselling; 3) Treatment of incomplete and unsafe abortions and complications; 4) Contraceptive and family planning services; and 5) Reproductive and other health services [8]*.*

Meanwhile, restrictive rules and regulations on abortion, as well as social and cultural norms stigmatize abortion and negatively affect uptake of PAC [10]. Partly due to stigmatisation, PAC faces ethical challenges which affects PAC research [11].

In order to improve PAC access and quality, there has been increased research on barriers and challenges for PAC involving adolescents [10, 12]. PAC researchers focusing on adolescents often face a range of ethics challenges, commonly due to abortion being a controversial, sensitive and stigmatized topic in many settings, including in LMICs where abortion is legal [13–15]. While some studies have been conducted on ethics issues associated with PAC research [15, 16], a comprehensive review of the ethics issues raised in international literature on adolescent post abortion care research is lacking. This study intends to contribute to addressing this knowledge gap by reviewing available scholarly literature to systematically characterize the nature and extent of documented ethics challenges faced by adolescent post-abortion care researchers, as well as any available guidance.

## Methodology

Using a scoping review design informed by Arksey & O’Malley [17, 18], we systematically searched relevant electronic databases (PubMed, HINARI, Google Scholar), as of November 2016, for literature raising ethics challenges faced by researchers involved in PAC research with adolescents. The search also included identification of any available ethics guidance on relevant issues. The following search phrases and terms were used: “abortion research AND ethics”, “adolescent health research AND ethics”, “guidelines AND adolescent health research AND ethics”, and “guidelines AND abortion research AND ethics”. Criteria were developed to capture peer-reviewed literature, published in English, and describing research ethics issues or ethics guidance relating to PAC or SRH more broadly. Exclusion criteria were also applied to remove PAC literature that did not focus on adolescents, and SRH literature that did not describe PAC research involving adolescents.

**Fig. 1:**
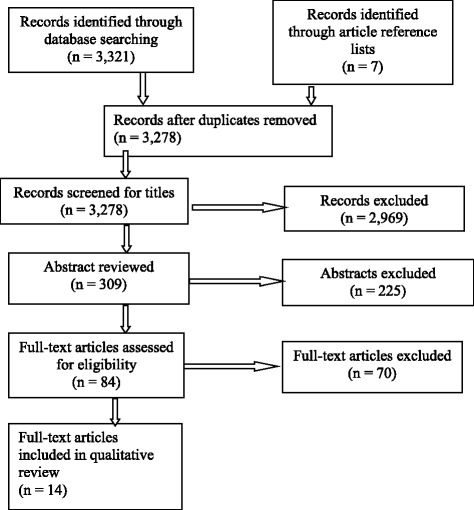
PRISMA flow diagram of scoping review [16]

Records initially identified through the search were screened to exclude duplicates, then titles were reviewed to exclude literature that was clearly unrelated to the subject matter. Irrelevant results were excluded and remaining literature was screened further (abstract) to determine applicability according to inclusion and exclusion criteria. Remaining records were reviewed (full-length) to identify a final list of papers meeting our criteria. We then reviewed the references of these articles to uncover any additional relevant literature that might have been missed through the initial search (Fig. 1).

### Analysis

Key ethics-related considerations raised in the literature were identified and grouped into themes and sub-themes following an inductive qualitative approach [18, 19]. The ethics domains (themes) and challenges (sub-themes) faced by researchers in adolescent PAC research, and suggested guidance on how to handle such challenges, were identified and coded inductively and iteratively by three individuals experienced in document review. Each person coded the literature applying themes and extracting challenges separately. Findings were compared and differences were reconciled through discussion until consensus was reached [18]. Once consensus was arrived on the ethics domains, challenges and suggested guidance on how to handle the challenges, the findings were tabulated.

## Results

A total of 3321 records were identified through the initial database search (HINARI - 31 records; Google Scholar - 458 records; PubMed - 2833 records). Forty-three duplicate entries were excluded. An additional 2969 records were removed following review of titles and 225 were excluded following review of abstracts. Of the 84 articles reviewed in their entirety, seven were identified as meeting inclusion criteria and an additional seven were identified from their references, yielding 14 articles for analysis (Fig. 1). No comprehensive, formal ethics guidelines on the topic were found, though some issue-specific guidance was identified embedded within the literature.

Table 1 lists the articles included in analysis, while Table 2 outlines the ethics domains, challenges and possible guidance for addressing these challenges that emerged from the review. The studies included in this review employed the following research designs: descriptive study design [1], cross sectional survey [1], secondary analysis on retrospectively collected data (1) and two studies adopted qualitative study designs (program evaluation and phenomenology). Four studies used longitudinal study designs while five studies were reviews. Five of articles focused directly and primarily on the ethical challenges associated with PAC research; the remaining nine commented secondarily on ethics issues within the context of PAC research. Seven provided guidance on potential means to navigate ethical issues in PAC research. Out of the 14 articles, two raised issues specific to adolescent PAC research while the rest focused on both adolescents and adults. Each ethics domain is further described below.

**Table 1:** PAC study details

Paper No	Authors; Year of publication	Title	Methodological approach	Main study focus/ objective
1.	Gipson JD, Becker D, Mishtal JZ, Norris AH; 2011 [15]	Conducting collaborative abortion research in international settings	Review of authors’ collaborative research experiences in conducting abortion-related studies using clinic- and community-based samples in five diverse settings (Poland, Zanzibar, Mexico City, the Philippines, and Bangladesh)	To share insights and lessons learned with new and established researchers to inform the development and implementation of abortion-related research
2.	Hess R; 2006 [13]	Postabortion research: Methodological and ethical issues	Phenomenology	To describe the ethical and methodological issues encountered during the study on post abortion
3.	Söderberg H, Andersson C, Janzon L SN; 1998 [14]	Selection bias in a study on how women experienced induced abortion	In each case information on socio-demographic characteristics, reproductive history and stated reasons for abortion was collected at the mandatory clinical visit prior to the abortion (Longitudinal study)	Using data from the mandatory preoperative visit, to compare those who agreed and those who refused to discuss their experience of the induced abortion and the care they had recived. Comparisions were made with regard to socio-demographic characteristics, reproductive history and stated reason for abortion
4.	Ringheim K; 1999 [16]	Ethical issues in postabortion care research involving vulnerable subjects	Review	To outline a set of principles developed by ethicists with specific reference to reproductive health that may help to meet the objectives of a rigorous ethical review called for in the case of vulnerable women during postabortion care
5.	Adler NE, David HP, Major BN, Roth SH, Russo NF, Wyatt GE; 1990 [20]	Psychological responses after abortion	Review	To document factors that shape responses after abortion
6.	Osur J, Baird TL, Levandowski BA, Jackson E, Murokora D; 2013 [21]	Implementation of misoprostol for postabortion care in Kenya and Uganda: A qualitative evaluation	Qualitative, program evaluation	Evaluate implementation of misoprostol for postabortion care (MPAC) in two African countries
7.	Reardon D; 1997 [22]	Limitations on postabortion research: Why we know so little	Review	To document the emotional aftermath of abortion
8.	Major B, Cozzarelli C, Cooper ML, Zubek J, Richards C, Wilhite M, et al.; 2000 [23]	Psychological responses of women after first-trimester abortion	Longitudinal study	To examine emotions, evaluations, and mental health after an abortion, as well as changes over time in these responses and their predictors
9.	Major B, Gramzow RH; 1999 [24]	Abortion as stigma: cognitive and emotional implications of concealment	Longitudinal study	To examine the stigma of abortion and psychological implications of concealment of their abortion
10.	Melkamu Y, Enquselassie F, Ali A, Gebresilassie H, Yusuf L; 2005 [25]	Assessment of quality of post abortion care in government hospitals in Addis Ababa, Ethiopia	Cross sectional survey	To assess the quality of health services with respect to postabortion care in hospitals in Addis Ababa, Ethiopia
11.	Evens E, Otieno-Masaba R, Eichleay M, McCarraher D, Hainsworth G, Lane C, et al.; 2014 [26]	Post-abortion care services for youth and adult clients in Kenya: a comparison of services, client satisfaction and provider attitudes	A descriptive, post-intervention study of PAC services was conducted in eight facilities in Central and Nairobi provinces	To examine receipt of PAC services by client age, client satisfaction and provider attitudes
12.	Prata N, Bell S, Gessessew a, 2013 [27]	Comprehensive abortion care: evidence of improvements in hospital-level indicators in Tigray, Ethiopia.	Secondary data analysis on retrospectively collected data	To assess trends in abortion-related morbidity indicators in referral hospitals
13.	Borges ALV, Monteiro RL, Hoga LAK, Fujimori E, Chofakian CBDN, & Santos OAD; 2014 [28]	Post-abortion contraception: care and practices	A longitudinal study of women hospitalized due to abortion in a public hospital	To analyze assistance regarding contraception methods received by women during hospitalization due to abortion, and contraceptive practices the month after this episode
14.	Wulifan, J. K., Brenner, S., Jahn, A., & De Allegri, M.; 2016 [29]	Scoping review on determinants of unmet need for family planning among women of reproductive age in low and middle income countries	Scoping review by employing mixed method approach.	To summarize the factors influencing unmet need among women in LMICs

**Table 2:** Ethics domains, challenges and guidance related to PAC research with adolescents identified in the literature

Ethics domains	Ethics challenges identified	Guidance within the literature
The role of local health providers, authorities and IRBs in approving PAC studies	-Difficulties in convincing local health providers or authorities to engage adolescents in PAC [16].	
Recruitment of adolescents	-Challenges in recruiting adolescents in the study [14, 15, 20–22].	Using several sources to recruit study participants [13].
	-Sampling from few clinics [20].	
	-Underrepresentation of women with unique characteristics such as those who find abortion stressful [20].	
	-Concealment of abortion affecting consent process [22].	
Informed consent	-Difficulties in seeking consent from relatives or parents of adolescents who are below the consent age [15, 16].	
	-Vulnerability of adolescents compromising ability to make decisions [16].	
	-Fear of losing access to health care affecting informed consent process [16].	
	-Inadequate guidance on how and when to involve “the community” in informed consent processes [16].	
Distribution of risks and benefits	-Selection bias such as having participants belonging to a particular group [20].	-Use of multiple methods may help reduce bias [13].
	-Difficulties with generalizability and validity of policy recommendations [14].	-Using several sources to recruit study participants [13].
		- Discussion of the risks and benefits of participation in the research [15].
		-Use research to foster positive attention, advocacy, support on abortion [15].
Handling of confidential information	-Maintaining confidentiality and privacy of data collected [13, 20, 23, 24].	-Avoided collecting personal identifiers- give reminder card which shows date and place of interview, and telephone number of the interviewer [15].
	-Disclosure of study participation very risky [20, 23, 24].	
	-Challenges in securing a conducive place for undertaking interviews [15].	-Creativity in identifying a secure space, which includes collecting data from an office away from the clinic [15].
	-Failure to properly secure the records of the patients after interviews [13, 15, 20, 23, 24].	-Ensure that dissemination of findings does not pose a risk by masking research sites, or collaborators, masking of clinic or community and providers [15]
	-Maintaining confidentiality and privacy of data may be challenging [13].	
	-Disclosure of study participation is very risky [20, 23, 24].	
Data collection: Participants and research staff/health providers	- Challenges with regard to data collection [13, 15, 16, 21, 22, 25–29].	-Training providers at all in capturing of data, including referral processes on PAC [27].
	-Difficulties in creating a non-prejudicial atmosphere [13].	-International researchers should always partner with local researchers [15]
	-Negative health provider attitudes and practices [15, 16].	-Understanding social norms [15].
	-Challenging in adhering to local norms [15, 16].	-The training and supervision of data collection staff and selecting staff with good attitude [15, 16].
	-Health workers wanting to be present during data collection [16].	
	-Role of the researcher who observes unethical health care practices [16].	
	-Paternalistic practices by health workers [16].	
	-Difficulties in managing emotional issues among adolescents [16].	
Data collection: quality of data	-The problem of social desirability bias [21], and under reporting [25, 26].	-The training and supervision of data collection staff and selecting of staff with experience working with the population [15, 16].
	-Incomplete records on the number of PAC services provided [27].	
	-Underreporting of abortion complications [27].	-Brief health workers after the study session and or/ bring up deficiencies in management meetings [16].
	-High dropout losses or attrition [22, 28].	
	-Cooperation is inconsistent and unreliable [22].	
	-Inadequate training among data collectors [16].	

### Role of local health providers, authorities and IRBs in approving adolescent PAC studies

Ringheim outlined important ethics challenges relating to convincing local health providers or authorities of the need to engage adolescents in both clinical and interview-based PAC research [16]. In some societies, where family planning matters are culturally conceived to only be appropriate for married people or adults, health providers or authorities may have difficulty allowing adolescents, especially those who are not married, to participate in PAC research or components of PAC research such as family planning [16]. The situation may be even more complex in contexts where abortion is illegal and there are reporting requirements. Difficulties in seeking authorization may therefore result in researchers abandoning PAC studies, which may affect the quality and availability of reproductive health services that address adolescent-specific health needs [16].

### Challenges in recruiting adolescents

Scholars highlighted the difficulties in reaching a proposed sample size of adolescents for both clinical and interview-based PAC research [14, 15, 20–22]. In contexts where abortion is illegal or stigmatized, researchers are reported to struggle in developing acceptable approaches for participant recruitment that do not put adolescents at risk of being reprimanded by their parents, stigmatized by society, or legally prosecuted [14]. Not only can recruitment challenges result in unrepresentative samples, they may also trigger selection bias where researchers deliberately select participants belonging to a particular group or those with similar characteristics due to accessibility [20]. In addition to selection bias, small samples may increase the possibility of exposing the identity of participants, thus putting them at risk. Moreover, poorly sampled data limits the generalizability and validity of policy or practice recommendations [14].

To partially address the problems that may arise due to small samples and selection bias, it has been recommended that researchers consider using multiple methods to triangulate data and minimize bias [13, 15, 20–22]. It has also been proposed that researchers consider using several sources to recruit study participants [13]. However, comprehensive guidance from published literature on how to handle recruitment challenges is lacking.

### Distribution of risk and benefits

Recruitment practices that emphasize maximization of efficiency may also result in ethical issues associated with the fair distribution of risks and benefits of the research. It is important for any type of research that particular populations not unjustifiably and disproportionately bear the burdens (or benefits) of research [13]. If PAC research unintentionally contributes to social stigmatization or physical harm to adolescents, this can have long-lasting effects, especially where limited social, economic and emotional support is available to participants. Certainly, PAC research involving adolescents involves, by necessity, a potentially vulnerable group, but the choice of study sites and sub-populations should reflect a diversity of contexts. Discussion of the risks and benefits of research participation is vital as it can help adolescents in making informed decisions [11].

### Informed consent

Ringheim suggested that obtaining valid informed consent can be a complex ethics challenge for PAC research involving adolescents [16]. Gipson et al. also explain that researchers may encounter difficulties with adolescents below the age of consent (usually below 18 years) feeling comfortable with researchers seeking consent from relatives or parents, as they may not want their parents or guardians to know that they were pregnant and that they had an abortion [15]. Thus, researchers may struggle to balance the need by adolescents for confidentiality and the demand for parental consent.

Other complexities regarding informed consent relate to the vulnerable status of adolescents who undergo abortions, particularly where abortions are illegal. Vulnerability may also be heightened by adolescents becoming pregnant before the culturally acceptable age or outside of marriage. It has been suggested that this state of vulnerability may compromise the ability to confidently make decisions regarding involvement in PAC research [16]. In addition, adolescents may feel obliged to participate in PAC research as they may feel that refusing to do so could affect their access to health care, particularly in cases where health providers are involved in the informed consent process [16].

How and when to involve “the community” in informed consent processes for PAC research involving adolescents is another complex issue raised in the literature. Challenges may arise in cases where some of the community representatives are opposed to abortion. In such cases, questions remain unanswered as to what obligations researchers have to seek community permission to conduct the research [16].

### Handling of confidential information

Quite a few articles suggested that maintaining confidentiality and privacy of data collected in PAC studies can be challenging because recruitment may take place through individuals who may, or may not, be required and trained to follow privacy promoting practices [13, 20, 23, 24]. Furthermore, when seeking consent from parents of an adolescent, researchers may disclose to parents or community members that the adolescent had an abortion [20, 23, 24]. Maintaining privacy of adolescents may also be difficult, especially in cases where adolescents are asked to come for repeated interviews and in situations where health workers (not on the research team) are involved in managing the information and scheduling appointments for interviews [12, 14]. Handling of private information can also be complicated by difficulties in finding a secure place to conduct interviews with adolescents. Health facilities in many LMICs are often particularly occupied and busy [15].

Privacy and confidentiality are crucial issues particularly in contexts where abortion is illegal and surrounded by social and cultural stigma, such that disclosure of study participation is very risky [20, 23, 24]. In such contexts, achieving privacy can be complex and may require some creativity and training on the part of researchers [16]. One study reduced such complexity by completely avoiding collection of personal identifiers; instead they gave each woman participating in the study a reminder card which showed the place where the interview would take place, date, time of interview and contact details of the interviewer for the women to call in case of delays or the need to cancel the interview [15].

Secluding adolescents for interviews should be done in such a way to avoid inadvertently generating suspicion, as this may trigger social and sometimes physical risk to the respondents [15]. It has been recommended that international researchers who may not be familiar with local norms and values always partner with local researchers in order to help ensure that cultural norms are understood [15]. While literature has highlighted these issues, it is not always clear how researchers can secure the best places for conducting interviews without negatively affecting the privacy of adolescents and generating contextual social suspicion.

### Data collection

#### Participant challenges

Multiple issues around effectively and ethically collecting data have been documented in the literature [13, 15, 16]. Ringheim documented difficulties in managing emotional issues among adolescents [16] and Hess discussed difficulties in creating a non-prejudicial atmosphere during interviews [13]. During interviews, some questions on abortion and care may make some adolescents recall negative experiences, which may trigger emotional responses. Questions have been posited in the literature as to when researchers have an obligation to counsel participants or refer them for counselling services once emotional problems are detected during interviews [16].

Hess stressed the challenges or struggles related to “creating a nonprejudicial atmosphere” [13]. Considering that abortion is a sensitive subject and has social, political, religious, and moral implications, personal views may influence researchers in their development of research questions and data collection tools, making it more likely that participants feel judged. During interviews, some researchers may integrate their personal views and beliefs about abortion and ask questions in ways that negatively affect respondents emotionally and psychologically. Limited training for researchers and research assistants in conducting studies on sensitive topics may contribute to this challenge [16]. Researchers may face other challenges in some communities where females, especially those who are married, are not expected to be interviewed without the partner being present [15].

#### Research staff and health provider challenges

Several health provider and health system charactersitics, such as health provider attitudes and the nature of services individuals and institutions are willing to provide, may pose ethics challenges in PAC research involving adolescents [15, 16]. In clinical settings, health workers may want to be present when research procedures are taking place [16]. This may be motivated in part by paternalistic practices and beliefs regarding what is thought to be best for their adolescent patients [16] or fear that adolescents may inform researchers about shortcomings in their health care. Permitting health workers to be present during data collection is likely to negatively affect participant privacy and may compromise data quality.

Managing situations in which researchers observe health providers interacting with patients in an unethical manner is another important challenge. For example, it has been suggested that providers may proceed to undertake clinical procedures without consent from the patient [16]. Ringheim further indicates that in some cases, providers do not properly attend to the management of post-abortion pain as they blame the patient for having resorted to unsafe abortions. Questions arise as to the role of the researcher who observes neglect or disrespect during care processes [16].

Ringheim suggested that researchers have an ethical obligation to brief health workers after the interview on the need to manage pain, address an unethical practice, or to bring up such deficiencies in regular briefings with management [16]. However, this may lead to researchers being denied further access if providers or facilities feel threatened. These ethical challenges are often compounded by limited training or limited exposure to best practices amongst providers for management of such challenges [16].

#### Quality of data

Ethical issues related to reporting or capturing quality data are prominent in the literature [13, 15, 16, 21, 22, 25–29]. Specific ethics-related challenges include failure to address social desirability bias - a tendency to answer questions in a way that will be viewed favorably by others [21] - and under-reporting of data in health facilities or institutions dealing with PAC [25, 26]. Researchers often face problems with incomplete records on the number of PAC services provided [27]. High dropout or attrition rates in PAC studies further complicates data collection and generalization of findings [22, 28]. Due to the sensitivity around discussing abortion, study participants responses to questions were sometimes inconsistent and unreliable [22]. Challenges in reporting data were also sometimes worsened by inadequate training among data collectors [16].

Suggested strategies for addressing some of these challenges include providing comprehensive training to PAC providers in capturing data and referral processes [27], ensuring adequate supervision of data collection staff, and selecting research staff with experience working with the population [15, 16].

Table 2 provides a summary of ethics domains mentioned in the studies.

## Discussion

This scoping review of the literature identified multiple ethics challenges faced by those who conduct research on PAC with adolescents. Ethics issues are reported to arise through the entire research process, from securing approval from IRBs and health providers to data collection and beyond.

The identified challenges may pose risks to study participants and undermine the development and uptake of PAC interventions. For example, inappropriate or incomplete consent processes in PAC research may expose study participants to risks they would not have otherwise agreed to take on [30, 31]. Threshold challenges, such as obstacles to obtaining study approvals, may lead researchers to abandon PAC studies, thereby compromising the attainment of public health goals. Abandonment of clinical and interview-based PAC studies may deny clinicians, health providers, and policy implementers needed evidence on the effectiveness, acceptability, and compatibility of PAC innovation in different environments.

Meanwhile, complications with sampling processes may result in systematic exclusion of relevant sub-populations from PAC research. This systematic exclusion may result in unfair distribution of benefits and burdens of research among adolescents [30]. Inadequate efforts to de-identify data and maintain participant confidentiality may result into social exclusion or stigmatisation of study participants. This can have long-term social, mental and physical effects on adolescents, especially in contexts where there is strong sense of kin and community [27, 28].

The review also revealed that despite the documentation of some ethics challenges associated with PAC research with adolescents, guidance on handling many of the issues is sparse. Formal guidance on how to address these ethics challenges could help researchers conduct studies in a more ethically appropriate manner [30, 32]. For example, addressing the ethics challenges related to informed consent, recruitment, and data collection is vital; left unaddressed, they can undermine study safety and respect for participants [31, 32]. Further research to clarify the nature and impact of evolving ethics obstacles in PAC research with adolescents is also needed.

Effectively addressing the ethics concerns related to informed consent, recruitment, and data collection may require use of innovative strategies to engage the community in PAC research with adolescents. Community engagement is likely to be vital as many of the ethics challenges identified in the literature are linked to community norms and values. Increased support for and acceptance of PAC at the community level may help adolescents or study participants make decisions without undue pressure from researchers, health workers or the community. There is need for conveyance of clear and unbiased information about what PAC research is and is not. Furthermore, community participation may increase trust between researchers and communities, potentially increasing the impact of the research itself [31, 32].

### Limitations

As with any literature review, it is possible that our search did not detect all publications that covered issues relevant to adolescent PAC research ethics, for example, due to inclusion only of studies conducted in English. We attempted to mitigate this limitation by reviewing different databases, conducting several types of searches, and reviewing references of articles found. The inclusion of journal articles, reviews, and books provided in-depth insight into the ethics challenges experienced by researchers conducting PAC research involving adolescents and possible guidance on how to address the challenges. The use of a multi-disciplinary team (with expertise in PAC research, bioethics, law, public health, and anthropology) in designing and conducting this review enriched the process, as the authors were able to provide input from various professional areas.

## Conclusion

Ethics challenges experienced by researchers involved in adolescent PAC research were identified in the literature at various levels. These included difficulties in seeking ethics committee approval to conduct PAC research, obtaining informed consent from adolescents, as well as observing unethical health care practices during clinical encounters and creating a non-prejudicial atmosphere during data collection. Suggested strategies for addressing some of these challenges included recruiting study participants through several sources, selecting study staff with experience working with the population, and briefing relevant health providers or managers on observed unethical practices after data collection. Sensitivity to potential unintended consequences of these strategies is also important. Promoting standards for appropriate data collection is crucial given the implications for the validity of results that inform PAC intervention development and policy. Overall, this review revealed that despite the documentation of challenges in PAC research with adolescents, guidance is limited and unconsolidated.

## Abbreviations

LMICs: Low- and middle-income countries
PAC: Post abortion care
SRH: Sexual and reproductive health
